# Social Epidemiology and Eastern Wisdom

**DOI:** 10.2188/jea.JE20120079

**Published:** 2012-07-05

**Authors:** Eric Brunner, Ayako Hiyoshi, Noriko Cable, Kaori Honjo, Hiroyasu Iso

**Affiliations:** 1 Department of Epidemiology and Public Health, University College London, London, United Kingdom; 2 Global Collaboration Center, Osaka University, Suita, Osaka, Japan; 3 Osaka University, Suita, Osaka, Japan

**Keywords:** social epidemiology, social science, social determinants of health, interdisciplinary research

## Abstract

Social epidemiology is the field of study that attempts to understand the social determinants of health and the dynamics between societal settings and health. In the past 3 decades, large-scale studies in the West have accumulated a range of measures and methodologies to pursue this goal. We would like to suggest that there may be conceptual gaps in the science if Western research models are applied uncritically in East Asian studies of socioeconomic, gender, and ethnic inequalities in health. On one hand, there are common concerns, including population aging and gendered labor market participation. Further, international comparison must be built on shared concepts such as socioeconomic stratification in market economies. On the other hand, some aspects of health, such as common mental disorders, may have culturally specific manifestations that require development of perspectives (and perhaps novel measures) in order to reveal Eastern specifics. Exploring and debating commonalities and differences in the determinants of health in Oriental and Occidental cultures could offer fresh inspiration and insight for the next phase of social epidemiology in both regions.

Social epidemiology aims to understand the wider determinants of health by using observational studies that measure an enlarged set of exposures. In addition to the usual downstream biomedical and behavioral risk factors, such studies include measurements of upstream factors that can be called “causes of the causes.”^1^ The growing interest in population research on aging motivates studies of a spectrum of novel age-related health outcomes, including vascular aging, functioning, and functional limitation.^2^ This methodology has generated much evidence that socioeconomic circumstances, living and working conditions, and social and psychological factors are strong influences on well-being and health over the life course. In policy terms, the health of a country’s population depends more on the ministries of finance, housing, education, employment, and environment than on the ministry of health—which would more accurately be referred to as the ministry of illness.^3^^,^^4^ Social epidemiology is science that supports the new public health movement^5^ and encourages interdisciplinary approaches that move outside the borders of conventional health promotion in search of effective interventions.

Research design is guided by theoretical models of the causes of the causes, and these models can be split into 2 contrasting groups. Materialist models emphasize income, employment, housing, and other “concrete” factors. Their strength is measurability. In contrast, psychosocial models seek explanations for social differences in health and well-being by studying social, family, and working relationships as well as beliefs and emotions. The strength of this level of explanation is that it may lead to a detailed understanding of the human experience of health and health inequality. Many studies have explored whether the materialist or psychosocial model is better at accounting for health inequalities within and between populations, and most of these were published between 1995 and 2005. The debate generated heat as well as light. Both levels of explanation have intellectual and empirical value, and their relative importance depends on the health outcome and context in question.^6^^,^^7^

The example of social capital shows that the distinction between materialist and psychosocial explanations can be exaggerated. Social capital has been measured in different studies by using several related components, including degree of social cohesion, number of social ties, and level of social trust.^8^ Both material and psychosocial advantage is gained when social capital is relatively high: there may be exchange of goods, loan schemes, and practical support of other kinds. There may also be emotional support at difficult times and a sense of belonging that enhances health and quality of life. Research in Japan has revealed such inter-relationships, eg, greater area income inequality was linked to a lower level of social trust and poorer self-rated health.^9^

Most evidence until now has been generated by research groups in the United States, United Kingdom, and mainland Europe, using Western population-scale studies.^6^^,^^10^^,^^11^ These centers have contributed to graduate training for an increasing number of groups of active East Asian researchers with the skills and interests to study the important question of social inequalities in health. It remains uncertain how health inequalities are evolving across East Asia in this new millennium.^12^^,^^13^ Japan remains at the top of the international life expectancy league table. Perhaps it also maintains low socioeconomic inequalities in health despite 2 decades of economic stagnation and a rate of relative poverty that is now similar to that in the United States and Mexico.^14^ If that were the case, Japan would be a prime example of a rich country maintaining excellent population health and managing to do so in a sustainable way.

One challenge for research on the social and economic determinants of health in East Asian countries is to understand how their particular social systems and cultures support or undermine health. In China, the prevalence of overweight doubled in women and tripled in men between 1989 and 2006, and there is evidence of emerging social inequality in this important health determinant.^15^ In South Korea, low education level has been linked with increased prevalence of metabolic syndrome in women, and this inequality appears to be growing in successive post-war birth cohorts.^16^ On the other hand, Japan’s frugal food culture has so far largely protected the population from the long march of the food corporations,^17^^,^^18^ except in Okinawa, where the dietary pattern is considerably westernized.^19^ The diversity in social trends across East Asian countries suggests that comparative studies would improve our understanding of how society influences population and individual health. In other words, there appears to be potential to study the considerable variation both in exposures and outcomes across the region. However, researchers in the region must determine whether there are important conceptual and methodological gaps in the science that has been developed in the Western context.

Some social dynamics are common to East and West. Increasing longevity brings the challenges of a new demographic that is the consequence of life expectancy at birth increasing at the rate of 3 months per year across a large number of countries.^20^ Although a great majority of the young old are able to live independently, surveys show the proportion that needs some social care tends to increase rapidly with age.^21^ The personal and collective costs in health-related quality of life and economic burden will be serious and difficult to manage if we ignore these at least partially avoidable problems. There is a lot to learn about healthy aging from studies in East Asia, and much would be relevant in the West.

As well as the shared concerns about the health of aging societies, there is another fundamental shift that interests social epidemiologists in the East and West, namely, the continuing trend toward gender equality within the family and in relation to the labor market. Age at first marriage is increasing and fertility is low, in part as a consequence of the desire of young Japanese women to be free of family demands at least until they have established a degree of economic and personal autonomy.^22^ An undesirable effect in the Japanese context is the high abortion rate: 22% of all pregnancies ended in induced abortion in 2002.^23^^,^^24^ One explanation may be that younger generations of women have a sense that the health effects of marriage are different for men and for women, ie, marital partnership results in fewer health benefits for women, whether they live in London or Osaka. The quality of the relationship is probably what matters. Positive psychosocial factors protect physical health, as shown in the inverse relation between social support and cardiovascular disease risk—more support, lower risk—in the East and West.^25^

Some aspects of population health are socially and culturally specific. This is most obvious in the tracking of vital statistics such as birth and death rates by country over time. Evidence on income inequality, life expectancy, and other health outcomes between and within countries suggests that distribution of material and other resources across a given society is a key determinant of health.^26^^,^^27^ Social stratification is an important issue in this respect because social epidemiology in part builds on the assumption that market economies generate social class hierarchies based on market or economic power, and that these are comparable.^28^^–^^30^ Further, a country’s system of social stratification is fundamental in the assessment of health inequalities and must be appropriately conceptualized and measured to capture the particularities of the society of interest. The labor market is a key dimension of social structure, and social scientists have discussed over several decades whether the Western concept of occupational social class is applicable to Japan.^31^^,^^32^ A social classification based on employment relations and status was found to detect similar variation and function of social classes in Japan, in comparison with Western countries.^31^^,^^33^ The research community is increasingly interested in social stratification; however, the Erikson–Goldthorpe classification—the theoretical basis of the UK National Statistics socioeconomic classification (NS-SEC)—has as yet been paid little attention, and the measure has not been applied by social epidemiologists in Japan to assess health inequalities.

With respect to social stratification, international comparison must be built on shared concepts and methods. In contrast, some health determinants would best be studied with culturally specific tools.^34^^,^^35^ Mental health is a particularly important dimension of health. The conventional, Western approach has proved to have weaknesses and thus a new understanding would be welcomed. Medication has long been the first-line treatment for depression in Europe and North America. However, it has been suspected for many years that drug treatment does not lead to improved outcomes except among those suffering from major and chronic depression. A recent expert review by the UK National Institute of Clinical Research confirmed this view and concluded that medication should no longer be the primary treatment for depression in the National Health Service (NHS). The headline advice in the detailed 2010 report tells doctors: “Do not use antidepressants routinely to treat persistent sub-threshold depressive symptoms or mild depression because the risk–benefit ratio is poor.”^36^

Research in East Asia may help to solve the widespread problem of chronic poor psychological health among adults. A Japanese study using the Beck Depression Inventory (BDI), which was developed in the United States, found that the BDI had similar validity in terms of factor structure in the United States and Japan, which implies that depression is a universal construct with universal symptoms and solutions.^37^ However, there is also a view that depressive symptoms may differ between Western and Eastern societies, particularly in their somatic manifestations (Ichiro Kawachi, personal communication). Somatic symptoms measured in the BDI are loss of energy, sleep problems, irritability, appetite problems, lack of concentration, tiredness, and sexual disinterest. Draguns mentions the greater separation between soma and psyche in Western culture.^34^ Related to this, there may be a lower level of cultural acceptance of depression as a largely mental disorder in East Asia.

These hypotheses suggest a need for studies using instruments developed by researchers who appreciate Eastern cultures, so as to inspire fresh thinking in the field of mental health. It could be that a difference in the pattern of depressive symptoms between East and West—which is not evident using the BDI—may be detectable using an instrument developed in the East. East Asian practices, perhaps with emphasis on social support networks, may work more effectively with mental distress than current antidepressant medications.^38^^,^^39^

If it is accepted that there might be culturally specific aspects of the social determinants of health, then it may be valuable to develop new constructs for use in social epidemiology. Such work would complement the extension of established methods, including measurement of socioeconomic position, to facilitate comparison of health inequalities in East Asian countries. It is likely that existing and new approaches are needed—combining development of newly validated psychosocial measures with validation of existing measures in China, Korea, Japan, and other East Asian countries—to understand relationships between social determinants such as strong community structures and levels of well-being in their respective populations.

Models of population health that are rooted in the cultures of East Asian countries may improve on models developed in the United Kingdom, for example, in their explanatory power for social inequalities in health outcomes. This is not to suggest that there is some mystical Oriental secret to health and longevity, but rather that the whole picture of East–West differences in social inequalities in health will not be captured if we favor Occidental constructs of psychosocial factors, well-being, and social position when such constructs are subject to major cultural and philosophical influences. Drawing on existing research models and methods is practical and has been productive during the first phase of social epidemiology in East Asia. Development of newly validated measures of social determinants inspired by East Asian researchers will surely be important for East and West in the second phase.
